# Free satellite image data application for monitoring land use cover changes in the kon ha nung plateau, vietnam

**DOI:** 10.1016/j.heliyon.2023.e12864

**Published:** 2023-01-07

**Authors:** Duy Ba Dinh, Dung Trung Ngo, Hoi Dang Nguyen, Hieu Huu Viet Nguyen, Ngoc Thi Dang

**Affiliations:** a Institute of Tropical Ecology, Joint Vietnam-Russia Tropical Science and Technology Research Center, No. 63, Nguyen Van Huyen Str., Cau Giay District, Hanoi, Viet Nam; b Forest Inventory and Planning Institute (FIPI), Vinh Quynh Commune, Thanh Tri District, Hanoi, Viet Nam; c University of Science, Vietnam National University, No. 334, Nguyen Trai Str., Thanh Xuan District, Hanoi, Viet Nam

**Keywords:** Natural forest, Mapping, Free satellite image, Policies, Kon ha nung

## Abstract

Remote sensing imagery is the most suitable tool for monitoring, managing, and evaluating land-use overlay fluctuations, especially forest cover for large areas. Free- and medium-resolution satellite imagery is a useful tool that allows scientific researchers and management organizations to monitor forest development in developing countries, such as Vietnam. In this study, we used SPOT 4 and Planet remote sensing data to assess land-use status fluctuations in the Kon Ha Nung Plateau area, Vietnam, between 2000 and 2021 (the overall accuracy was 90.52%, Kappa value = 0.89). The results showed that from 2000 to 2010, the rate of natural forest loss in this area was 0.32%/year, of which, more than 6500 ha were converted to other uses. Between 2010 and 2021, the rate of natural forest loss gradually decreased (0.09%/year) instead of fluctuating between different types of land use. The area of forests, perennial crop land, and annual crop land tended to increase from 2000 to 2010; however, from 2010 to 2021, the area of plantation forests decreased markedly, while the area of perennial crop land and annual crop land continued to expand. The analysis of the policies on forest management, exploitation, and protection was applied locally, to explain the causes of the change in spatiotemporal aspects of the types of land-use cover in the Kon Ha Nung Plateau. Restoring forest areas during 2010–2021 initially improved effectiveness in forest management and protection. Furthermore, the results provide a better understanding of the current position and role of the government apparatus, cadres, and ethnic minorities in socioeconomic development associated with forest protection and development on the Kon Ha Nung Plateau. The results of this study can help managers monitor annual forest-cover fluctuations based on free remote sensing imagery to reduce both the cost of management and surveying, yielding relatively accurate results.

## 1 Introduction

Forests and forest ecosystems cover a large part of the Earth and account for the majority of biological productivity on continents. The structure and function of forests are regulated by a wide range of factors, including the physical environment, plant growth, demonstration processes, biochemical cycles, and disturbance mechanisms [1]. Tropical forests cover approximately 10% of the total surface area of the Earth; nonetheless, tropical forests play a crucial role in the global carbon and water cycles and are home to more than half of the species globally [2]. Moreover, globally, approximately 1.6 billion people depend on forests to varying degrees, with 350 million living in or near jungles [3]. Forests are a source of food, fuel, medicinal herbs, handicraft materials, and income [[4], [5], [6]]. Economically, forests provide a variety of goods and services to humans, bringing income and other forest products to local people [7].

Forests and forest ecosystems are threatened on a global scale, especially in the tropics, because of agricultural deforestation, logging, hunting, wildfire, climate change, and other anthropogenic activities [2,[8], [9], [10]]. Tropical forest biodiversity is increasingly threatened by deforestation and various other factors, which lead to forest degradation [4]. The annual rate of biodiversity decline, mainly in tropical rainforests, is approximately 17,500 ha/year [11]. To reduce forest loss and promote conservation, the 10.13039/100004420United States has considered the framework REDD+ (Reducing Emissions from Deforestation and Forest Degradation and + mechanism for enhancing carbon stocks and conservation and sustainable management of forests) and forest conservation funding from developed and developing countries [12,13].

Most tropical forests are not properly managed [14] for the following reasons: (i) Lack of financial benefits from the application of forest management based on policies, as well as the unwillingness of the consumer to pay a high price for timber from well-managed forests or certified operations; (ii) Government policies to improve forest management are mostly not guaranteed to be fair from stewards to forest users, and lack of serious government commitments in enforcing forestry regulations, ensuring clear ownership and the right to use, poor management capacity in reducing forest abuse; and (iii) An incomplete understanding of the benefits of the application of forest management improvements, the lack of staff with professional training, and limited technical expertise. In some areas, community-managed forests are less degraded than those that are government-managed, which supports the management of common resources [15]. Forestry policies are paramount in forest management as they guide the actions of foresters or natural resource managers at a certain location in a landscape [16]. Management policies and project programs related to forests are often direct causes of fluctuations in large-scale forest areas. In Iran, the government introduced many forest conservation policies through reforestation, forestry, and community forestry projects. However, for various reasons, forest management in Iran has still not succeeded in conserving forests, and the development plans of the government were not applied according to the program [17]. As reported by Nambiar, the Indonesian government granted concessions to domestic and international companies to convert millions of hectares of degraded forestland for reforestation of timber and oil palms [18]. Similarly, in Vietnam, government and local policies for restoring reforestation and greening trees helped restore some of the national forest cover rates [19]. However, unreasonable policies, such us restoring reforestation and greening trees, and weak management can cause loss of forest area and degradation. Additionally, the conflict of interest between the rights of the forest management community to enjoy forest resources and those of the groups exploiting these resources has also led to loss of forest area and degradation [20]. Additionally, in Vietnam, income from forest environmental services are gradually becoming popular among the forest management communities; however, they have not yet become a stable source of income for the local people, limiting long-term forest environmental services [21]. Therefore, assessing the fluctuation of tropical rainforest cover and analyzing local land-use policies are important bases for management, planning, conservation, and development of one of the most important ecosystems globally.

One of the most common methods in forest cover monitoring is the application of remote sensing and Geographic Information System (GIS) technology [[22], [23], [24]]. Particularly, the use of remote sensing images is very popular and is widely applied [[25], [26], [27], [28], [29], [30]]. For tropical forests, in case of no data or a lack of latest data on the spatiotemporal distribution of forest cover, satellite remote sensing data have been widely used for current status mapping and forest fluctuations [31]. Other applications related to unmanned aerial vehicles (UAVs) have also been used in forest resource research [[32], [33], [34]]. Forest status maps are the most suitable tools for displaying the content and status of forest vegetation. Forest mapping is based on the nomenclature defined by the Food and Agriculture Organization [35]. Many forms of multiscale and temporal satellite imagery have been used to establish forest cover status maps and assess forest ecosystem fluctuations [[36], [37], [38], [39]]. Assessing forest cover fluctuations and monitoring changes in forest states at spatiotemporal scales forms the scientific basis of forest resource management, conservation, and development [[40], [41], [42], [43], [44], [45], [46]].

Medium-resolution free remote sensing images have been used extensively to assess forest fluctuation overlays with high accuracy. In a previous study, Sentinel-2 images were used to establish a highly accurate forest cover assessment and classification map (98.3% and 94.8%, respectively) [47]. Free multi-source remote sensing imagery, including that with Sentinel-2A, Sentinel-1A, and Landsat-8, combined with topographic data obtained from eight forest classification studies in Wuhan, China, achieved 82.78% accuracy [48]. In the Daxing Mountains of China, SPOT-5 and RADARSAT-2 satellite imagery combined with forest-type classification achieved an accuracy of up to 88%, which is a 12% improvement compared to that using only SPOT data [49]. Many algorithms have been applied to improve the accuracy of maps and to classify overlay types more accurately. Currently, the random forest algorithm is widely used to classify objects in forest maps and overlays. Breiman et al. developed a random forest algorithm for the first time in 2001 to increase the accuracy of forest object classification [50]. Random forests are a combination of tree predictors in a manner where each tree depends on the values of a random vector sampled independently and with the same distribution for all the trees in the forest. Since then, many studies have used the random forest algorithm to create overlay maps and classify forests based on remote-sensing imagery. Sentinel-2 and Landsat-8 satellite imagery used for forest classification-based analysis using random forest algorithms provide better accuracy for maps, as in Brazil, with a K-factor of approximately 0.9 [39]. In a study in Gabon, Sentinel-2 satellite imagery combined with a random forest classifier yielded an overall accuracy that ranged from 83.4 to 97.4% [37]. Additionally, this method was also applied to the classification of tree species with a relatively high overall accuracy (84.5%; Kappa value 0.73) [38] or an overall accuracy (82%) in Austria [51]. Generally, for large areas, free satellite image sources, such as WorldView, Sentinel, SPOT, Planet, and Landsat, are suitable tools for temporal forest status mapping to monitor forest development. They are also effective data sources for researchers in areas with developing economies and low investment costs for forest resource management and protection, such as Vietnam.

The Kon Ha Nung Plateau in the Gia Lai Province, Vietnam, was registered as the World Biosphere Reserve at the 33rd session of the UNESCO International Coordinating Council of the Human and Biosphere Program (MAB-ICC) in Abuja, Nigeria, on September 15, 2021. The core areas of this biosphere reserve are the Kon Ka Kinh National Park and Kon Chu Rang Nature Reserve. The Kon Ka Kinh National Park and Kon Chu Rang Industrial Park are relatively intact forests that are highly diverse in vegetation characteristic types: tropical enclosed forests evergreen broad-leafed; coniferous evergreen rainforest; evergreen forests with broad leaves; coniferous evergreen forests characteristic of the forest ecosystems of the Central Highlands provinces, with many unique features and outstanding and unique characteristics [52]. Additionally, a carpet of shrubs, grasslands, agricultural land, and residential areas are present. In the period of 2000–2021, the changes in forest cover were mainly related to the forest conservation and developmental policies of the Government of Vietnam as well as the local authorities.

The objective of this study was to map forest-cover fluctuations for the period of 2000–2021 based on remote sensing images (GIS), impact analysis, assessment of the effectiveness of policies for protection, forest development, and changes in land-use structure in the Kon Ha Nung Plateau area in Vietnam.

## 2 Materials and methods

### 2.1 Study area

**Fig. 1:**
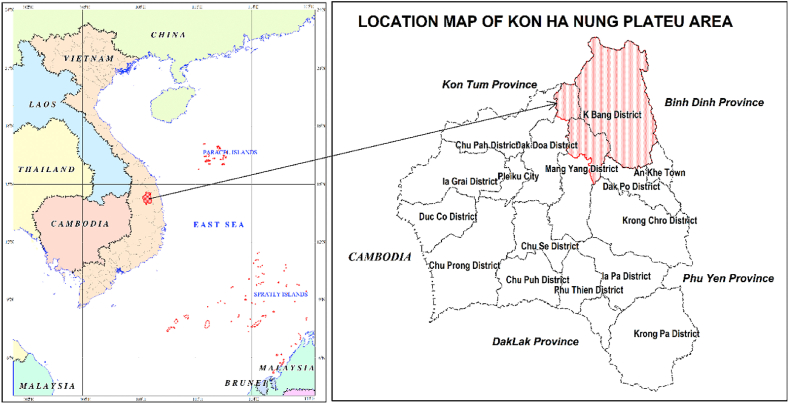
Map depicting the location of the Kon Ha Nung Plateau area.

Kon Ha Nung Plateau is a plateau in Gia Lai province, spread over the area of K’bang and An Khe districts, and was recognised by UNESCO as a world biosphere reserve on September 9, 2021. Kon Ha Nung Plateau Biosphere Reserve has a total area of more than 413,500 ha. The core area of this biosphere reserve is Kon Ka Kinh National Park and Kon Chu Rang Nature Reserve. The buffer zone is a forest area stretching from Chu Păh district to Kbang district (Gia Lai), bordering Kon Ray district, Kon Plong (Kon Tum province), Ba To district (Quang Ngai province), An Lao district, and Vinh Thanh (Binh Dinh province) (Fig. 1).

The plant ecosystem here is mainly a type of moist tropical evergreen forest in low and medium mountains [53]. Part of the area is a semi-evergreen forest or picket forest. The flora here is highly biodiverse, rated A for international importance, with thousands of species. In particular, there are many rare plant species included in the Red Book of Vietnam and the world such as *Hopea hainanensis*, *Aquilaria crassna, Anoectochilus setaceus*, *Dalbergia cochinchinensis*, *Pterocarpus macrocarpus Kurz*, etc. The animal ecosystem here also has thousands of species. Many species are in the group that should be preserved in the Red Book of the world such as *Pygathrix cinerea*, *Nomascus annamensis*, *Buceros bicornis*, *Heliopais personatus*, *Pterocarpus macrocarpus*, *Aquilaria crassna*, etc [54].

In the buffer zone of the Kon Ha Nung Plateau, there are many ethnic minority communities, mainly Bahnar and Jrai people. These are communities with unique cultures; in particular, their lives are associated with the Central Highlands Gongs Cultural Space, which is inscribed by UNESCO on the list of intangible cultural heritage [54].

### 2.2 Materials

**Fig. 2:**
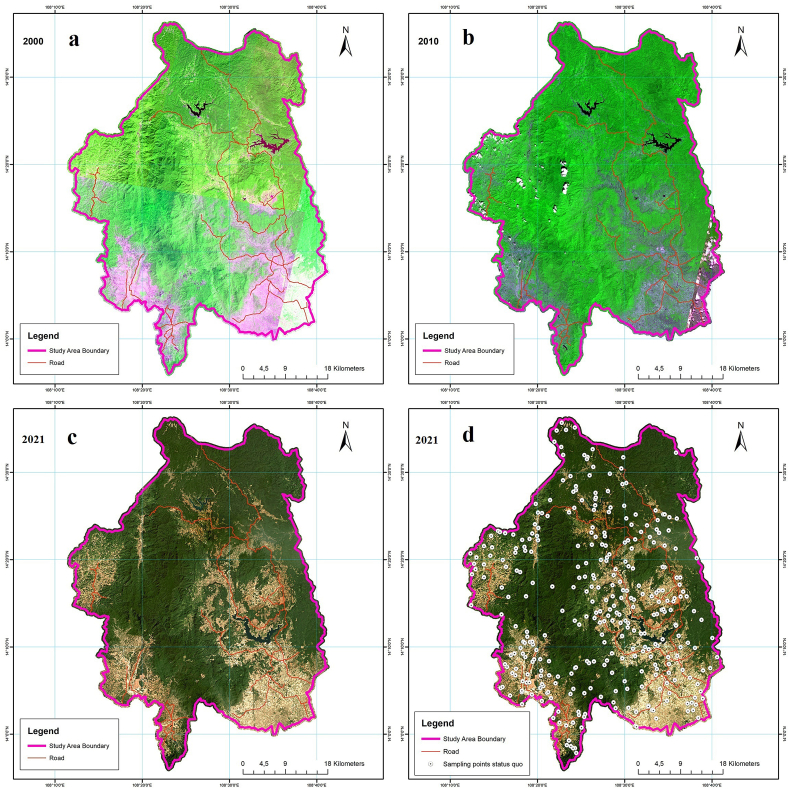
Temporal remote sensing images of 2000, 2010, 2021 (a, b, c) and 2021 photo key sample test points (d) in the Kon Ha Nung Plateau area.

In the Kon Ha Nung Plateau area, January–March is the end of the dry season, with very little rain. Satellite imagery at the study site during this period was virtually unaffected by cloud factors as well as other atmospheric factors, which is consistent with the quality and timing of image acquisition. On the basis of temporal selection when satellite imagery had the lowest cloud cover during this period, we used SPOT-4 satellite imagery data from 2000 (Fig. 2a), 2010 (Fig. 2b), and Planet (2021) (Fig. 2c) to map the land-use cover of the Kon Ha Nung Plateau, Gia Lai Province, Vietnam (Table 1).

**Table 1:** Specifications of remote sensing images.

Date	Sensor	Scene ID	Image bands	Resolution
February 14, 2000	SPOT-4	42783210002140330182I	Pan (610–680 nm) Green (500–590 nm) Red (610–680 nm) Near-infrared (780–890 nm),	Panchromatic: 10 m × 10 m Multispectral: 20 m × 20 m
March 02, 2010	SPOT-4	42783211003020258271I	Blue (0.455–0.525 μm), Green (0.530–0.590 μm), Red (0.625–0.695 μm), Near-infrared (0.760–0.890 μm)	Panchromatic: 10 m × 10 m Multispectral: 20 m × 20 m
January 30, 2021	Planet	20200430_235834_1013	Blue (0.455–0.525 μm), Green (0.530–0.590 μm), Red (0.625–0.695 μm), Near-infrared (0.760–0.890 μm)	All bands: 4,7 m × 4,7 m

### 2.3 Methods

#### 2.3.1 Establishment of land-use cover status maps

##### 2.3.1.1 Image analysis to map the status of land-use cover

eCognition software is specialized for processing and interpreting satellite images [55][56]

The image-interpreting key template (MKA) used for 2021 photo prediction was included in the analysis on Collect Earth. Next, 80% of the total number of templates was assigned a status after fragmentation. The remaining 20% of MKA (330 samples) was used to verify the scene and assess the accuracy (Fig. 2d). The same was applied to the 2000 and 2010 MKA sets, which introduced the MKA sets of each year into the Earth software and used Google Earth photos to assign statuses for interpretation.

First, the image lock for image interpretation will be included in the eCognition software to conduct identification according to the image properties. Next, the multiresolution segmentation algorithm will rely on the uniform criteria of the image lock template to merge pixels based on similarity [57]. Here, the similarities between the photo pixels are defined as the spectral characteristics of the photo [58]. The multiresolution segmentation algorithm allows for locating objects and the boundaries between them. In the next step, objects with a small area will be merged into objects with a larger area. At this step of combining small areas, the process was optimised to minimise the heterogeneity of objects based on the 2 parameters n and h (n is the size of a circle, and h is the arbitrary definition of heterogeneity). As a result, adjacent pairs of similar objects will be merged together to form a larger circle. The process stops when the smallest growth exceeds the threshold defined by the rate parameter [59].

Spectral or color heterogeneity was described by the following formula:

(1) $$h=\sum_{c}w_{c}\sigma_{c}$$

Accordingly, the heterogeneity of the value h is calculated as the ratio between the circumference of a circle and the square root of the total number of pixels that make up that circle (called n). The calculation formula is described as follows [60]:

(2) $$h=\frac{l}{\sqrt{n}}$$

##### 2.3.1.2 Categorized by the random forest algorithm

According to Genuer (2020), the method of assigning status after image segmentation performed by eCognition Developer software using random forest is a newly developed technique. The random forest classification method, which is a comprehensive machine learning method for classification, regression, and other tasks that functions by building many decision trees during the training period and providing layers that are the method of the layers (classification) or the average prediction (regression) of each tree [61].

Accordingly, the random forest algorithm was described as follows [50].

Consider an ensemble of classifiers *h1(X)* and *h2(X)*, $h_{k}$ *(X)*, and with the training set drawn at random from the distribution of the random vector Y, X, we define the margin function as:

(3) $$mg\left(X,Y\right)=av_{k}I\left(h_{k}\left(X\right)=Y\right)-\underset{j\neq Y}{\underbrace{\max}}av_{k}I\left(h_{k}\left(X\right)=j\right)$$

where, *I* (·) is the indicator function. The margin measures the extent to which the average number of votes at X and Y for the correct class exceeds the average vote for any other class. The larger the margin, the greater is the confidence in the classification. The generalization error is given by:

(4) $$PE^{*}=P_{X,Y}\left(mg\left(X,Y\right)<0\right)$$

where, the subscripts X and Y indicate that the probability is greater than that in the X and Y spaces.

#### 2.3.2 Verification of classification results

Checking the accuracy of the image interpretation results is the next step after the preliminary image guessing results have been obtained. At this step, each subject needs a minimum of 10 points to verify the accuracy of the field survey results. If the accuracy is less than 75%, it is necessary to re-check the image interpretation process and redefine the image interpretation key to improve the accuracy of the process [62]. The Kappa value (K) is regarded as the most effective tool for evaluating the accuracy of image interpretation results [63]. Accordingly, to calculate K, it is necessary to build a table of error matrices with the deviation of the matrix based on the data of rows and columns [64] and define the following values.

1. Overall accuracy assessment
2. User accuracy assessment
3. Producer accuracy assessment

The formula defines the K value as follows:

(5) $$K=\frac{N\sum_{i=1}^{r}X_{ii}-\sum_{i=1}^{r}\left(X_{i+}-X_{+i}\right)}{N^{2\;}-\sum_{i=1}^{r}\left(X_{i+}-X_{+i}\right)}$$

where, *r* is the number of columns in the image matrix, $X_{ii}$ is the number of pixels observed in row *i* and column *i* (on the main diagonal), *i* receives values from 1 to *r*, $X_{i+}$ is the total pixels observed in row *i*, $X_{+i}$ is the total pixels observed in column *i*, and *N* is the total number of pixels observed in the image matrix.

The usual K value will range from 0 to 1. For a K value less than 0.4, the accuracy is rated as low; if the K value ranges from 0.4 to 0.8, the accuracy is rated as average; if the K value is greater than 0.8, this is considered high accuracy, according to the U.S. Geological Survey.

### 2.4 Mapping land-use cover fluctuations

To map the fluctuations in land-use cover for the periods 2000–2010 and 2010–2021 in the Kon Ha Nung Plateau area, we first determined the current land-cover attributes for each contour of the vegetation maps for the periods 2000–2010 and 2010–2021. Accordingly, seven types of land covers, namely natural forest, plantation forest, industrial crop land, shrub, grassland, bare land, agricultural land, rural resident land, and water surface were considered.

**Fig. 3:**
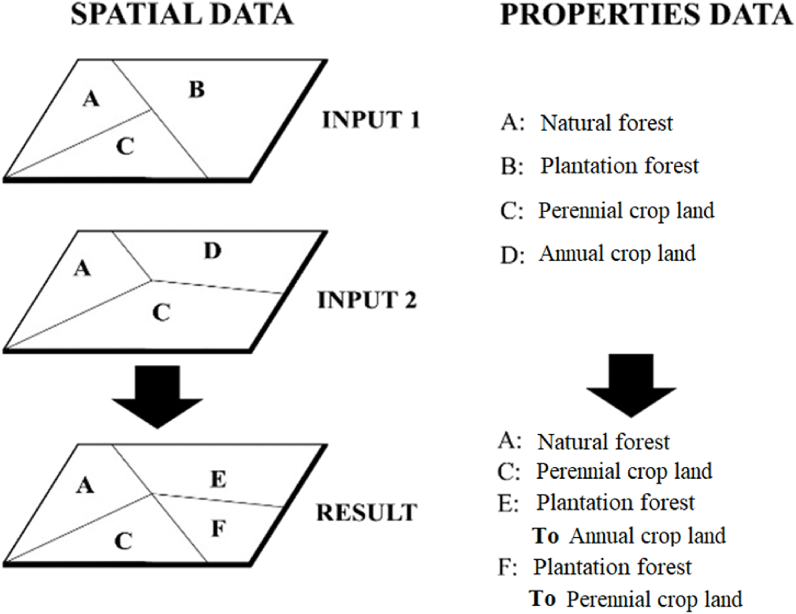
Schematic illustrating the process of using GIS to establish a land cover change map.

Second, we overlaid two layers of maps that had homogenized attributes of land cover according to the corresponding years of the vegetation-change map, based on the intersection algorithm of ArcGIS 10.8 software. The overlay of the maps was modeled, as shown in Fig. 3.

Thus, the results created a layer of land-use fluctuation map showing the cover states between the two moments.

## 3 Results

### 3.1 Results of image classification and disinterest

Based on the results of the examination of photo guessing samples and formulas for calculating coefficient K (Equation (5)), an overall accuracy index matrix table was established for the current map of land-use cover in the Kon Ha Nung Plateau area in 2021 (Table 2).

**Table 2:** Error matrix to solve the image of land-use cover in the Kon Ha Nung Plateau area.

		Ground Reference							
		Natural forest	Plantation forest	Perennial crop land	Annual crop land	Shrub, grassland, and bare land	Rural resident land	Water surface	Total
**Classification**	Natural forest	**94**	2	2	1	1	0	0	**100**
	Plantation forest	1	**45**	3	1	0	0	0	**50**
	Perennial crop land	1	2	**47**	0	0	0	0	**50**
	Annual crop land	0	0	1	**34**	4	1	0	**40**
	Shrub, grassland, and bare land	0	0	0	1	**37**	1	1	**40**
	Rural resident land	0	1	0	2	2	**25**	0	**30**
	Water surface	0	0	0	0	1	0	**19**	**20**
Total	96	50	53	39	45	27	20	**330**	
User Accuracy (%)	97.92	90.00	88.68	87.18	82.22	92.59	95.00	**90.51**	
Producer Accuracy (%)	94.00	90.00	94.00	85.00	92.50	83.33	95.00	**90.55**	
**Overall Accuracy (%)**	**90.53**								

According to Table 2, the overall accuracy index for 2021 is 90.53%, corresponding to K = 0.89. Accordingly, the water surface objects are the most easily recognizable, with a total of 19/20 samples interpreting images in accordance with reality. Perennial cropland and plantation forest area can be misinterpreted, with a total of 5 out of 100 samples of these two subjects having been misinterpreted. The structure of the satellite image of these two subjects has many similarities, resulting in inaccuracies duirng image interpretation. Annual croplands were also observed to suffer from relatively high rates of decoding to shrubs, grasslands, and bare land (4/40 samples). Some natural forest dissection samples were also misinterpreted as plantation forests (2/100 samples) and perennial cropland (2/100 samples). The type of land in rural resident areas also had a high rate of misinterpretation of annual crop land, shrub, grassland, and bare land (2/30 samples misinterpreted).

Overall, all seven land-use types included in the image analysis had an accuracy of over 80%. Natural forests and water surfaces had the highest user accuracy (97.92% and 95.00%, respectively), in which, the map accuracy of the water surface type was the highest of all types (95%). Shrub, grassland, and bare land had the lowest user accuracy (82.22%), while rural resident land had the lowest producer accuracy (83.33%).

### 3.2 Mapping of the status of land-use cover in the kon ha nung plateau area

**Fig. 4:**
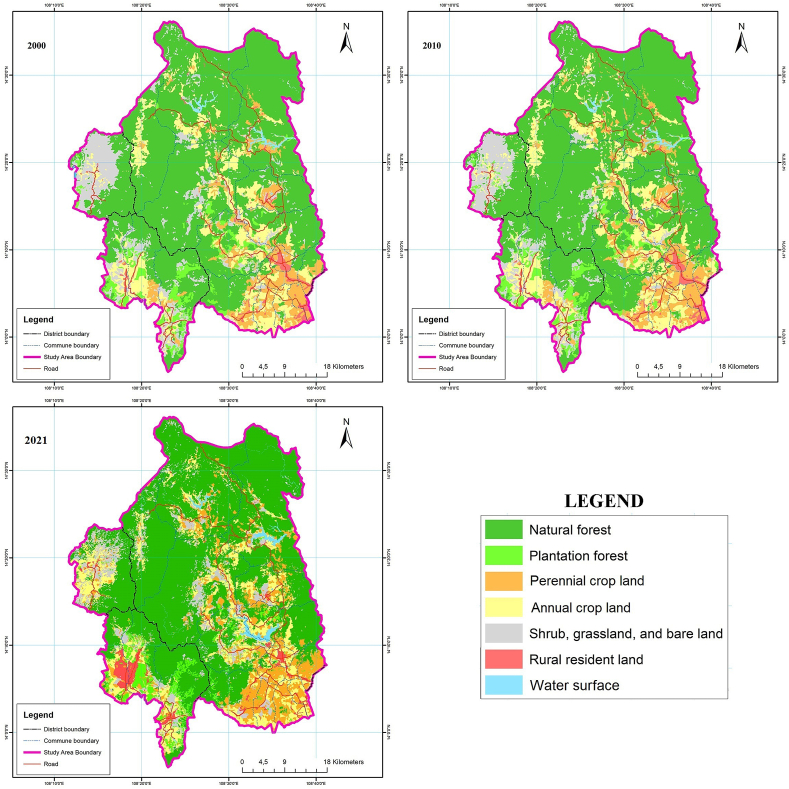
Map of the current status of land use cover in the Kon Ha Nung Plateau in 2000, 2010, and 2021.

Based on the process of interpreting temporal remote sensing images with the multiresolution segmentation algorithm (Equation (1(1) and (2(2)), the random forest algorithm (Equation (3(3) and (4(4)), and evaluating the results of image classification, overlay maps of the Kon Ha Nung Plateau area were established three times: 2000, 2010, and 2021 (Fig. 4).

According to Table 3, the area of natural forests in the Kon Ha Nung Plateau accounted for the largest area of all types of land use in 2000, 2010, and 2021 (over 150,000 ha in the entire period of 2000–2021). However, the annual rate of forest loss in 2000–2010 was relatively high (0.33%/year) compared to that in 2010–2021 (0.09%/year). The area of annual crop land during the entire period of 2010–2021 was the second largest compared to that of other types of land use, which tended to increase gradually in this period, with a rate of increase of 0.84% and 0.30%/year in the periods 2000–2010 and 2010–2021, respectively. Similarly, regarding the type of land in rural residential land, the rate of area increase over the year was also relatively high, with 0.67%/year in the period 2000–2010 and 6.82%/year in the period 2010–2021. Contrarily, shrub, grassland, and bare land tended to decrease gradually, with a decrease of 1.07% and 1.74% per year in 2000–2010 and 2010–2021, respectively. The area of the plantation forest type was relatively volatile during the entire period of 2000–2021. During 2000–2010, the plantation forest area increased by nearly 2000 ha, reaching a rate of 1.90%/year. However, during 2010–2021, the plantation forest area decreased by more than 3300 ha, with an average decrease of 2.42%/year. The area of perennial crop land in the Kon Ha Nung Plateau area in 2000 reached over 20,000 ha, with a sharp increase in the period 2000–2010 (increased to 3000 ha, equivalent to 1.46%/year). By the end of 2010–2021 period, the area continued to increase by nearly 6500 ha, with an average annual increase rate of 2.50%/year.

**Table 3:** Statistics on the area of land use in the Kon Ha Nung Plateau in 2000, 2010, and 2021.

Unit: ha							
Land-use cover	Area in year			2010–2000		2021–2010	
	2000	2010	2021	area change (ha)	area change (%)	area change (ha)	area change (%)
Natural forest	156,723	151,613	150,102	−5110	−0.33	−1511	−0.09
Plantation forest	10,417	12,394	9094	1977	1.90	−3300	−2.42
Perennial crop land	20,558	23,552	30,027	2994	1.46	6475	2.50
Annual crop land	31,042	33,647	32,534	2605	0.84	−1113	−0.30
Shrub, grassland, and bare land	25,489	22,763	18,409	−2725	−1.07	−4354	−1.74
Rural resident land	4027	4296	7520	269	0.67	3224	6.82
Water surface	1745	1735	2315	−10	−0.06	580	3.04
**Total**	**250,000**	**250,000**	**250,000**				

**Fig. 5:**
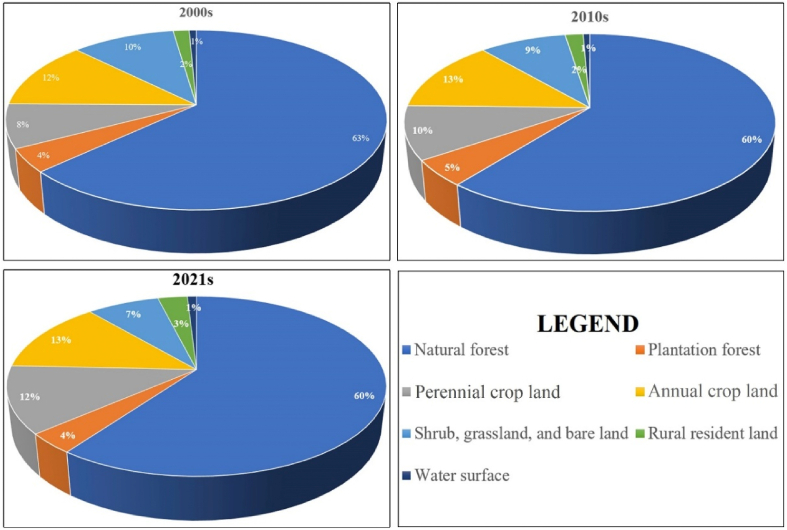
Pie charts depicting the area structure of land-use cover in the Kon Ha Nung Plateau during 2000–2021.

In 2000, the proportion of natural forest area in the Kon Ha Nung Plateau area was the highest among all types of land use, accounting for 63% of the total area (Fig. 5). The proportions of annual cropland and shrub, grassland, and bare land ranked 2nd and 3rd, respectively (12% and 10%, respectively). Simultaneously, the area of plantation forests and land in rural residential areas was relatively small. Similar to 2000, the area structure of land-use types in 2010 and 2021 did not change significantly. Particularly, the structure of the natural forest area decreased to 60% in both years, and the area of annual cropland was higher than that in 2000, at 13% for both years. Similarly, the total areas of shrubland, grassland, and bare land also declined compared to 2000, falling to 9% in 2010 and 7% in 2021. The proportion of plantation forests was relatively stable, ranging from ∼4 to 5% during 2000–2021. The proportion of rural resident land over the years has remained consistent from 2000 to 2021 and 2021, at 2–3% of the total natural area of the Kon Ha Nung Plateau (Fig. 5).

### 3.3 Assessment of land-use cover fluctuations in the kon ha nung plateau area

Based on the map of the status of land-use cover of three years, i.e., 2000, 2010, and 2021, two maps of land-use cover fluctuations were established, and the fluctuation of the area of the forest cover in the Kon Ha Nung Plateau area through two stages i.e., 2000–2010 and 2010–2021, were assessed.

**Fig. 6:**
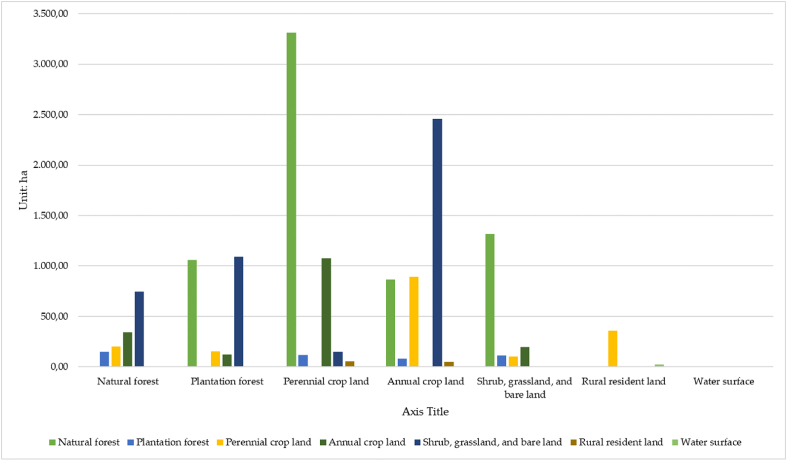
Bar plot showing the area converted between types of land use in the Kon Ha Nung Plateau during 2000–2010.

According to Fig. 6, at the end of the 2000–2010 period, more than 6500 ha of the natural forests in the Kon Ha Nung Plateau area was lost, which was mainly converted into plantation forests, perennial crop lands, and shrubs, grasslands, and bare land (1058.88 ha, 3309.51 ha, and 866.23 ha, respectively). However, nearly 1500 ha of other types of land were converted into natural forest areas, mainly shrubs, grasslands, bare land, annual croplands, and perennial croplands. The area of perennial crop lands was also highly volatile during this period, with more than 1700 ha of its area converted into other types of land use. Particularly, nearly 900 ha of perennial crop land was converted into land for annual crop lands. Contrastingly, natural forest area contributed more than 3300 ha, and over 1000 ha of annual crop land were also converted into perennial crop land. During 2000–2010, the total areas of shrubs, grassland, and bare land also varied, and nearly 4500 ha were converted to other types of land use, mainly annual crop land, plantation forests, and natural forests (2459.12, 1093.95, and 747.80 ha, respectively). More than 1300 ha of natural forests have been converted to shrubs, grasslands, and bare lands. The type of land in the countryside and the water surface did not fluctuate significantly during this period. The strongest transformation of the two types manifested in just over 350 ha of perennial crop land that has been converted into rural resident land.

**Fig. 7:**
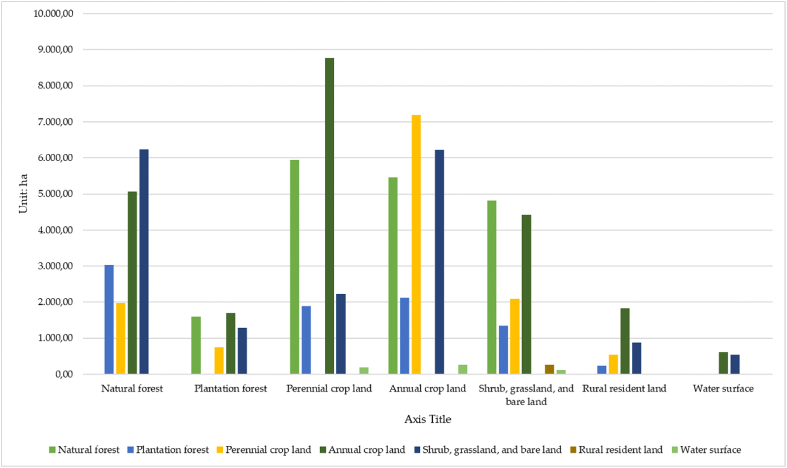
Bar plot showing the area converted between types of land use in the Kon Ha Nung Plateau during 2010–2021.

The land-use types in the Kon Ha Nung Plateau area showed strong fluctuations from 2010 to 2021 (Fig. 7). Particularly, nearly 18,000 ha of natural forests were converted into other types of land use, mainly perennial croplands, annual crop lands or shrubs, grasslands, and bare lands (5949.55, 5467.90, and 4810.81 ha, respectively). However, more than 16,300 ha of other types of land use were converted to natural forest lands, such as 6233.53 ha of shrubs, grasslands, and bare lands, 5067.23 ha of annual crop lands, and 3027.47 ha of plantations. The plantation forest area also drastically changed, mainly being converted to natural forests, annual crop land, perennial crop land, shrubs, grasslands, and bare lands (a total of more than 8600 ha was converted).

Contrastingly, more than 5300 ha of other types of land were converted to plantation forests, mainly annual crop lands, natural forests and shrubs, grasslands, and bare lands. Annual crop land was one type of land use that drastically altered during this period. The overall area during this period showed little change, but the conversion of this type was very large. Specifically, nearly 22,400 ha of annual crop land were converted to different types of land use, mainly perennial crop lands (8777.24 ha), natural forests (5067.23 ha), shrubs, grasslands, and bare lands (4422.33 ha). Contrastingly, more than 21,200 ha of other types of land use were converted to annual croplands, but the largest were perennial crop land, shrubs, grasslands, bare lands, and natural forests.

**Fig. 8:**
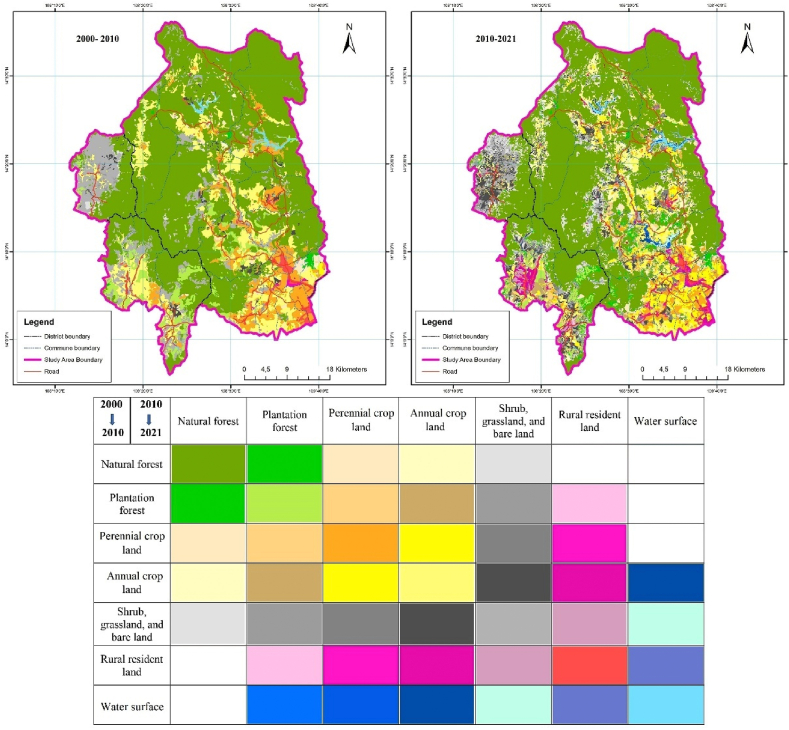
Map of land-use cover fluctuations in the Kon Ha Nung Plateau area during 2000–2010 and 2010–2021.

The type of land in rural residents also tended to increase in area during 2010–2021, which was mainly converted from annual crop land (1824.42 ha). However, some areas of land in the countryside were also converted to shrubs, grasslands, and bare lands. The water surface area also expanded fairly largely, mainly being converted from annual crop land and shrubs, grasslands, and bare lands (increased to more than 1000 ha, concentrated largely in the Ka Nak lake area of the K'bang district) (Fig. 8).

### 3.4 Effect of forest protection policies and development on land-use area fluctuations in the kon ha nung plateau area

During 2000–2021, the government of Vietnam and the Gia Lai People's Committee implemented many policies to strengthen the management and support forest protection in the Central Highlands, Gia Lai Province, and the Kon Ha Nung Plateau area. These policies are stated as follows.

1. Decision No. 07/1999/CT-UB dated May 4, 1999, of the Gia Lai People's Committee on a number of urgent measures to prevent the exploitation, processing, transportation, sale, and use of illegal wood and forest products, which include cutting down forest trees as pillars, destroying forests, encroaching on forestland to grow coffee. Accordingly, for deforested areas, encroachment on forestland to grow coffee or food crops and trees, apart from handling crimes against forest protection and development, it is necessary to immediately destroy areas of coffee plantation, food crops, or other illegally planted trees, forcing violators to replant forests.
2. Decision No. 03/2002/QD-UB dated January 3, 2002, of the Gia Lai People's Committee on the conversion of 14 forests into forest protection management boards. Accordingly, forest area exploited for resources are converted into protected forest management boards, tasked with managing forest protection, developing forest capital, and using forests under management in accordance with the regulation on three types of forests (protection forests, production forests, and special-use forests).
3. Decision No. 04/2002/CT-UB dated March 13, 2002, of the Gia Lai People's Committee on strengthening urgent measures for forest fire prevention and fighting. Accordingly, it is necessary to further strengthen the PCCCR work, limiting to the lowest area of forest burned in the area, along with strengthening propaganda, dissemination, legal education, and the policy of the state on forest fire prevention. Forest fire prevention measures should be developed in a timely manner.
4. Decision No. 87/2002/QD-UB dated November 1, 2002, of the Gia Lai People's Committee on the establishment of the Forestry Development Sub-Department under the Department of Agriculture and Rural Development. Accordingly, the forest protection management apparatus has clear functions and tasks in the management, protection, and development of forest resources.
5. Decision No. 10/2003/CT-UB dated May 28, 2003, of the Gia Lai People's Committee on strengthening urgent measures to protect and develop forests. Accordingly, strengthening measures to prevent and effectively address illegal forest exploitation, illegal deforestation, and other violations of forest protection management in the area; creating conditions for local people to have enough productive land and instructing them on intensive production, strengthening the extension of agriculture and forestry for ethnic minorities living near forests, developing rice areas, gradually limiting the area of rice, guiding the transformation of livestock crop structure to suit each locality, and gradually stabilizing life for people. Additionally, forest management, poaching, and protection should be strengthened.
6. Decision No. 106/2003/QD-UB dated October 1, 2003, of the Gia Lai People's Committee on the promulgation of temporary regulations on beneficial forest securities under Decision No. 178/2001/QD-TTg. Accordingly, the authorities at all levels develop securities plans, make security dossiers, create security in the field, form security contracts, and benefit policy; applied to forests, the Forest Protection Management Board is assigned a pilot plan for forest securities delivery. Thus, it is possible to ensure the harmonization of interests between the state and the person directly protecting the forest, the economic benefits of forests, and the benefits of protecting the ecological environment between immediate and long-term benefits. Use forest resources reasonably to offset costs and generate income for forest-keepers.
7. Decision No. 26/2007/QD-UBND dated March 1, 2007, of the Gia Lai People's Committee on the conversion of Ha Nuong Forest into Ha Noi Forestry Company. Accordingly, the Ha Noi Forestry Company will have greater function and power in the management and protection of forests in the area.
8. Decision No. 18/2007/QD-TTg dated February 5, 2007, of the Prime Minister of Vietnam on approving Vietnam’s forestry development strategy for the period of 2006–2020, which requires the People's Committee at all levels to strengthen the management, protection, and development of forest resources, including the Kon Ha Nung Plateau area.
9. Decision No. 18/2007/CT-UBND dated December 26, 2007, of the Gia Lai People's Committee on strengthening measures to prevent forest fires in the dry season of 2008.
10. Decision No. 75/2015/ND-CP dated September 9, 2015, by the Prime Minister of Vietnam on mechanisms and policies for forest protection and development, which is associated with the policy of rapid and sustainable poverty reduction and support for ethnic minorities from 2015 to 2020. Accordingly, economic households in difficult mountainous areas and ethnic minorities in the Kon Ha Nung Plateau area are prioritized to carry out tasks related to forest management and protection, receiving forest securities, with a support amount of VND 400,000/ha/year. Additionally, added reforestation support is funded by a budget of VND 1,600,000/ha/year in the first 3 years and VND 600,000/ha/year for the next 3 years. Support of 5,000,000 VND for 10, 000, 000/ha to buy seedlings, fertilizers, and other costs for reforestation. Supported rice at a rate of 15 kg/gun/month or in corresponding money, but not more than 7 years. Additionally, there are a number of other loan support policies to promote the economy for disadvantaged households and ethnic minorities.
11. Decision No. 38-CTr/TU dated March 29, 2017, of the Gia Lai People's Committee on strengthening the Party's leadership on forest management, protection, and development in the whole province, which emphasizes the role and importance of two special-use forests in the Kon Ha Nung highland area, Kon Ka Kinh National Park, and Kon Chu Rang Nature Reserve.

Additionally, the authorities at all levels and management boards of special-use forests and protection forests in the area of the Kon Ha Nung Plateau implemented propaganda programs for people in the area of forest protection and forest allocation, improving the important role of forest resources for local people, especially ethnic minorities.

## 4 Discussion

### 4.1 Land-use cover maps of the kon ha nung plateau area based on temporal remote sensing imagery

Temporal remote sensing is a suitable tool for monitoring and assessing fluctuations in forest resources and other types of land-use cover [31,65], which is the basis for management, supervision, conservation, and sustainable development of forest resources for managers and local authorities [66].

The method of using high-resolution remote sensing imaging provided accuracy when mapping the land-use cover in the Kon Ha Nung Plateau area (overall accuracy = 90.53%; K = 0.89). This was highly accurate when compared to the results of certain previous studies that used satellite imagery Landsat-8 and Sentinel-2 (overall accuracy = 75%; K = 0.65) [67], those that used an amalgamation of Sentinel-2A, Sentinel-1A, Landsat-8 and Digital Elevation Model imaging data in Wuhan, China, with an overall accuracy of 82.78% [48], or those that used Sentinel-1 and Sentinel-2 satellite imagery in Spain and Brazil (the intermediate K value was only 0.59–0.83 for Sentinel-2 and 0.28–0.72 for Sentinel-1 images) [36]. The mapping accuracy in our study is also higher than that of studies that used low-resolution multitemporal Landsat imagery data for mapping the eastern Sundarbans, Bangladesh, from 1989 to 2019 to understand mangrove dynamics over a period of 30 years; the overall accuracies of Landsat TM (1989), TM (2014), and L8 OLI (2019) were 80%, 82.85%, and 84.28%, respectively [23], and in Sundarbans, the use of Landsat satellite imagery between 1975 and 2020 for mangrove cover classification resulted in an accuracy of 84.8–90% [68]. The combination of temporal remote sensing images and topographic data also contributed to increased map accuracy, such as the use of consecutive Landsat satellite imagery in a time series with an overall accuracy of up to 90.52%; whereas combining topographic data improved the overall accuracy by 92.63% in Vinton County, southeastern Ohio [69]. When both WorldView-2 and airborne LiDAR data were used in Toronto, Canada, the overall accuracies obtained using ResNet-18, ResNet-34, ResNet-50, and DenseNet-40 were 90.9%, 89.1%, 89.1%, and 86.9%, respectively [70].

In this study, we enhanced the mapping accuracy by classifying forests using random forest algorithms. This method significantly enhanced the accuracy of land-cover mapping [47]. Sentinel-2 and Landsat-8 satellite imagery were used for analysis based on forest classification using random forest algorithms that provided better mapping accuracy, as in Brazil, with a K coefficient of ∼0.9 [39], which is the same as that of our study. In a study in Gabon, Sentinel-2 satellite imagery combined with random forest taxons resulted in an overall accuracy of 83.4–97.4% [37]. Additionally, this method has been applied to the classification of tree species with a relatively high overall accuracy (84.5%; K value 0.73) [38] and an overall accuracy of 82% in Austria [51].

However, for free satellite images of medium resolution, mapping accuracy still has certain errors despite being used along with the random forest algorithm and the eCognition software. For improving errors and enhancing map accuracy, conducting field surveys and collecting sufficiently large image interpretation keys is necessary to increase the accuracy of the image analysis process. Additionally, medium-resolution carpet images should only be applied for sufficiently large areas and scales that are suitable to the mapping scale.

The discrepancy in the process of interpreting photos of the Kon Ha Nung Plateau area is mainly on the types of annual croplands and other soils, empty land without plants, in accordance with the study by Fekri et al. [71]. These types have certain structural similarities in satellite image data and are prone to misinterpretation. Additionally, interpretive patterns between plantations and perennial crops have a relatively large rate of misinterpretation, as they have many structural similarities in the satellite imagery. For minimizing errors in the interpretation process, including a field process of verifying the image interpretation key sample set is necessary.

### 4.2 Fluctuations and causes of land-use cover fluctuations in the kon ha nung plateau from 2000 to 2021

During 2000–2021, the government and local authorities in Vietnam implemented many policies to strengthen the management, protection, and development of forest resources in the Kon Ha Nung Plateau area. These policies have had specific impacts on the fluctuation of the area of land-use covers in the region, which is typical of the fluctuation of the area of natural forests, plantation forests, annual planting land, and perennial planting land.

During 2000–2010, the areas of all types of plantations, perennial planting land, and annual tree land increased significantly. Decision No. 10/2003/CT-UB on the recovery of illegally encroached areas for industrial and agricultural crops is the cause of the area fluctuation in the types of crop lands and annual crop lands, shrubs, grasslands, and bare lands. Additionally, in the conversion of Ha Nung Forestry to Ha Nung Forestry Company, the deployment of reforestation and industrial trees in the areas of vacant land, and grassland in the management area have contributed to minimizing the area of shrubs, grasslands, and vacant land, instead of perennial planting lands, such as *Coffea canephora*, Macadamia, citrus plants, and plantation forests. Although there were many policies on forest protection and development, the forest protection work during 2000–2010 were not highly effective. This is reflected in the decrease in forest area during this period, with the rate of forest loss reaching 0.32%/year, equivalent to more than 5000 ha of natural forest being converted into different types of land use.

The forest-loss rate decreased significantly between 2010 and 2021. For achieving the above effect, policies to strengthen forest protection management (decision No. 18/2007/QD-TTg, decision No. 38-CTr/TU) and implementing forest protection securities (decision No. 75/2015/ND-CP) are crucial. Particularly, the policy on forest protection and support for ethnic minority people to stabilize their livelihoods is considered highly suitable for the Kon Ha Nung Plateau area, where up to 80% of the population comprises ethnic minorities. Therefore, the total area of natural forests in the Kon Ha Nung Plateau area was relatively stable during this period. Particularly, the natural forests in two special-use forest areas on the Kon Ha Nung Plateau (Kon Ka Kinh National Park and Kon Chu Rang Nature Reserve) remained stable. This is the result of many policies to reorganize the management apparatus of forest management boards and policies to strengthen the protection of special-use forest resources in the Kon Ha Nung Plateau area. However, the fluctuation of area between natural forests and types of land use in this period is still relatively high and concentrated in the areas of production forests next to villages. Decision No. 75/2015/ND-CP also supports seedlings, fertilizers, and supplies for ethnic minorities, helping to restore areas of shrubs, grasslands, and bare lands in the area into areas of plantation forests and perennial crop lands during 2010–2021.

During 2000–2021, many policies to strengthen the management, protection and development of forests were enforced for the Kon Ha Nung Plateau. These policies have partially reduced the loss of natural forests before the 2000s; however, they have not yet had a clear effect. Among them, Decision No. 75/2015/ND-CP is considered the most important, contributing to stabilizing the livelihoods of ethnic minorities in the locality, linking the management of forest protection with the village community, and supporting people in planting forests to recover forests. This is also the general policy of the government of Vietnam for the management, protection, and sustainable development of forest resources nationwide. The culture of indigenous peoples, their understanding of nature, and their skills to adapt to the environment related to forests make an important contribution to the conservation of natural resources and socioeconomic development associated with the lives of residents in mountainous areas [7].

## 5 Conclusions

In this study, current land-use status maps and land-use overlay fluctuation assessments based on free medium resolution remote sensing imagery were established for the Kon Ha Nung Plateau area for the period 2000–2021. We propose that free remote sensing images of medium resolution may be used to monitor annual land-use fluctuations on large-scale areas.

Our study has a certain limitation. Medium-resolution remote sensing images are only suitable for large-scaled areas, such as districts, provinces or regions that are even larger. For small areas, even for areas approximately 100–200 ha, relatively higher resolution remote sensing imaging data are needed. Drones may be used for monitoring land-use mantle fluctuations.

Our study results may provide a basis for formulating policies to protect and develop forest resources appropriately and for planning the development of local forestry industry in a timely and effective manner. In the future, with the advancement of remote sensing technology, increased remote sensing image resolution can be attained, thereby providing an effective tool for managers to monitor annual land-use overlay fluctuations with increased accuracy.

## Author contributions

Dung T.N. and Duy B.D. conceived and designed the experiments; Duy B.D., Dung T.N., and Hoi D.N. performed the experiments; Dung T.N, Duy B. D, and Hieu H·V.N. analyzed and interpreted the data; Hieu H·V.N. and Ngoc T.D. contributed materials; Duy B.D., Dung T.N., and Hoi D.N. wrote the paper**.**

## Declaration of competing interest

.
